# Augmenting small biomedical datasets using generative AI methods based on self-organizing neural networks

**DOI:** 10.1093/bib/bbae640

**Published:** 2024-12-10

**Authors:** Alfred Ultsch, Jörn Lötsch

**Affiliations:** DataBionics Research Group, University of Marburg, Hans – Meerwein - Straße, 35032 Marburg, Germany; Institute of Clinical Pharmacology, Goethe - University, Theodor - Stern - Kai 7, 60590 Frankfurt am Main, Germany; Faculty of Medicine, University of Helsinki, Haartmaninkatu 8, 00014 Helsinki, Finland; Fraunhofer Institute for Translational Medicine and Pharmacology (ITMP), Theodor-Stern-Kai 7, 60596 Frankfurt am Main, Germany

**Keywords:** machine learning, generative algorithms, data science, biomedical data, self-organizing maps, artificial neurons, data generation

## Abstract

Small sample sizes in biomedical research often led to poor reproducibility and challenges in translating findings into clinical applications. This problem stems from limited study resources, rare diseases, ethical considerations in animal studies, costly expert diagnosis, and others. As a contribution to the problem, we propose a novel generative algorithm based on self-organizing maps (SOMs) to computationally increase sample sizes. The proposed unsupervised generative algorithm uses neural networks to detect inherent structure even in small multivariate datasets, distinguishing between sparse “void” and dense “cloud” regions. Using emergent SOMs (ESOMs), the algorithm adapts to high-dimensional data structures and generates for each original data point *k* new points by randomly selecting positions within an adapted hypersphere with distances based on valid neighborhood probabilities. Experiments on artificial and biomedical (omics) datasets show that the generated data preserve the original structure without introducing artifacts. Random forests and support vector machines cannot distinguish between generated and original data, and the variables of original and generated data sets are not statistically different. The method successfully augments small group sizes, such as transcriptomics data from a rare form of leukemia and lipidomics data from arthritis research. The novel ESOM-based generative algorithm presents a promising solution for enhancing sample sizes in small or rare case datasets, even when limited training data are available. This approach can address challenges associated with small sample sizes in biomedical research, offering a tool for improving the reliability and robustness of scientific findings in this field. Availability: R library “Umatrix” (https://cran.r-project.org/package=Umatrix).

## Introduction

Obtaining sufficient data to conduct valid biomedical research is often challenging [1–3]. Reasons for small sample sizes in research data include limited resources for data collection, the rarity of a disease, or the intention to limit sample sizes in preclinical research as a measure of animal welfare. For example, acute promyelocytic leukemia (APL) accounts for less than 4% of all leukemia cases in Germany [4], making comparative gene expression studies with other types of leukemia difficult. Small sample sizes are also common not only in several other rare genetic disorders, such as cystic fibrosis, but also in early-stage drug trials for diseases such as Alzheimer’s [5] and personalized medicine approaches in oncology [6], where patient-specific factors limit the number of participants. Another reason for small datasets may be that the classification of multivariate data is often costly and time-consuming and requires experts in the field, leading to sparse labeling (e.g. assignment of a clinical diagnosis) of the data [7].

Sparse data in biomedical research can lead to poor reproducibility and challenges in translating results from animal models to clinical applications [8–12]. Recent approaches to address this issue include multicenter study designs [13], combining data from different sources [14], and meta-analyses. Generative artificial intelligence (AI) has emerged as a potential solution, with algorithms such as Gaussian mixture models (GMMs) being explored [15], as well as more complex approaches such as generative adversarial networks (GANs) [16], latent Dirichlet allocation [17], Boltzmann machines, and others [18–21]. These methods often use probabilistic approaches, sampling from given distributions to generate synthetic data. However, their effectiveness depends on the ability to accurately formulate the underlying distribution of the sparse dataset.

**Figure 1:**
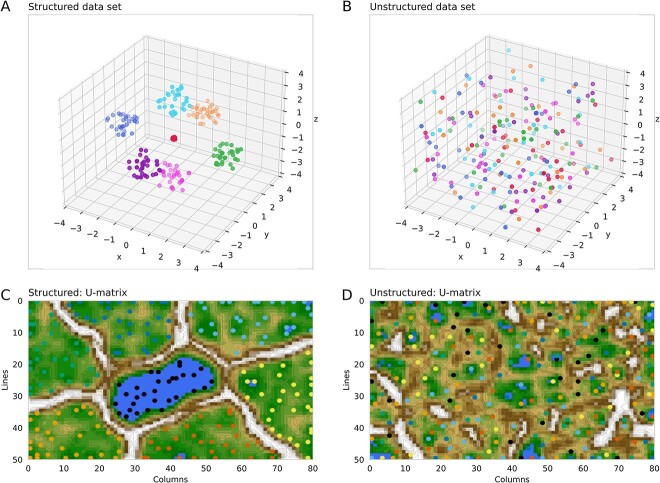
“Structured” versus “unstructured” data sets and the suitability of ESOMs to capture structure. (A) The synthetic “Hepta” data set [25] comprising *n* = 212 data points evenly distributed across *k* = 7 classes within well-defined and well-separated data-rich regions arranged as seven spheres in a cube with edge coordinates [−4, 4]. This represents a “structured” data set. (B) In the same cube, *n* = 212 data points are located at random, representing an “unstructured” data set. (C) The 3D U-matrix visualization of the distance-based structures of the “Hepta” data set following the projection of the data points onto a toroid grid of 4000 neurons. The dots represent the BMUs, i.e. neurons on the grid that carried a data vector most similar to a data vector for a sample in the data set. The U-matrix visualization is colored as a topographic map, with brown “heights” and green “valleys” with blue “lakes.” Watersheds indicate borderlines between different clouds separated by white “mountain ridges.” BMUs belonging to different clouds are colored green or bluish. (D) The projection of the unstructured data set onto the ESOM grid resulted in a structure-less U-matrix representation that provided no hints at a class structure. The structured data set (A) is clearly reflected in the similar seven-class structure on the U-matrix (C), while the unstructured data set (B) merely resulted in a structure-less U-matrix (D), demonstrating the suitability of ESOM to capture the underlying structure or lack thereof in the data. The figure was created using Python version 3.11.5 for Linux (https://www.python.org) with the seaborn statistical data visualization package (https://seaborn.pydata.org [88]).

We propose a novel generative algorithm based on the well-known self-organizing maps (SOMs) of artificial neurons [22] to increase the sample size in small data sets. The emergent SOM (ESOM) approach captures intrinsic data structures through cooperative neuron interactions [23]. It allows us to visualize the inherent structure in the data [23, 24] (Fig. 1). The ESOM-based algorithm then generates additional data structurally equivalent to the original dataset, without introducing artifacts or spurious structures. This generative AI method seems to be particularly effective for small biomedical datasets and overcomes the limitations of conventional generative techniques.

## Methods

### Basic considerations

#### Concept of structure in multivariate data sets

**Figure 2:**
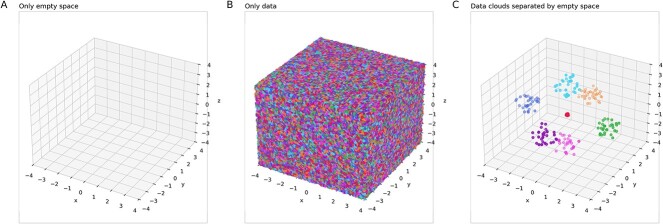
Visualization of the concepts of empty data space versus clouds of data. Assuming that the cubes drawn below represent the entire data space, cube A represents a completely empty data space with no data points, i.e. the distance $d$ between data points approaches infinity $\left(d\to \infty \right)$; cube B represents a data space completely saturated with data points, where each point touches its neighbors, leaving no gaps $\left(d\to 0\right)$; and cube C represents a more typical scenario in which the data space consists of clusters or “clouds” of data points separated by empty regions, reflecting the natural grouping and spacing commonly observed in real-world data sets $\left(0<d<\infty \right)$. The scenario in (C) is the main scenario addressed in this report on structure-preserving data generation. The figure was created using Python version 3.11.5 for Linux (https://www.python.org) with the seaborn statistical data visualization package (https://seaborn.pydata.org [88]).

Multivariate data sets can be divided into two different types of regions in (hyper-)space. There are regions of empty space (void), where virtually no data points are located, and regions containing densely arranged data points, referred to as data clouds (Fig. 2). Figure 1A shows this in more detail, along with the ESOM-based data structure detection method used here. Within these data clouds, the distances between neighboring data points (topological neighbors) are significantly smaller than the distances in the regions of empty space. Consequently, the density, i.e. the number of data points per unit volume, is low in the voids and high within the data clouds. On the other hand, if the empirical multivariate data are homogeneous or uniformly distributed within the variable ranges, there is no discernible structure in the data (Fig. 1B). However, measured experimental conditions, such as treated versus untreated or healthy versus sick patients, in a data set can alter the shape and/or location of the data clouds.

Importantly, the above-presented concept of structure in data does not require that the structure of the data clouds or their location need to be described by a specific statistical or mathematical probabilistic model. However, if such a probabilistic model is available, it can and should be used to analyze the data and generate additional data. In the following, we describe a method for generating additional data for multivariate data sets that exhibit the structural features described above. This approach ensures that the generation of new data neither destroys the existing structures nor introduces spurious structures into the data.

#### Probabilistic model-based versus model-free data generation

**Figure 3:**
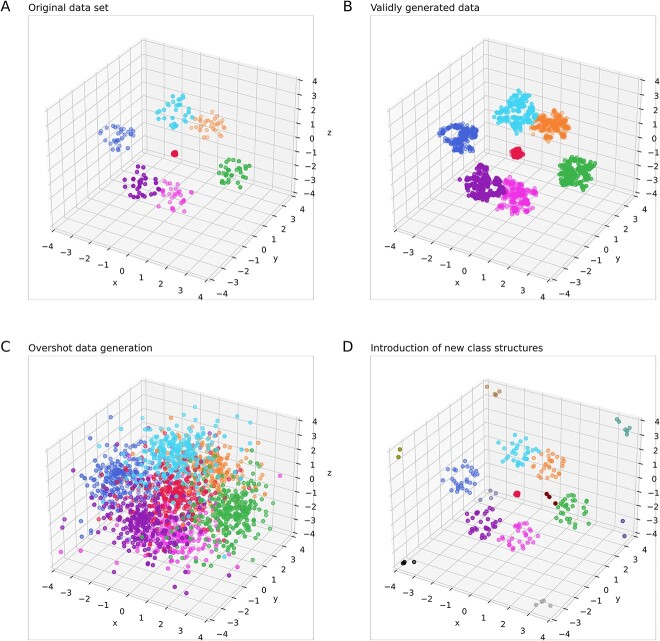
Augmentation of a small data set using generative AI. The goal is to augment the small data set while maintaining the underlying data structure and class boundaries, without introducing artifacts or new, unsupported classes. (A) The synthetic “Hepta” data set [25] comprising *n* = 212 data points evenly distributed across *k* = 7 classes within well-defined and well-separated spherical data clouds. (B) The desired result is the generation of data points that are not identical to the original points but well preserve the general structure of the data set. The generated data could have been acquired in the experiments provided that the sample size was larger. (C) In contrast, the generated data should not destroy the original structure of the data set by generating data points that break the original class limits or blur the initial class structure of the data. (D) Similarly, the generated data should not introduce new classes for which no indication of their existence was available in the original data. The figure was created using Python version 3.11.5 for Linux (https://www.python.org) with the seaborn statistical data visualization package (https://seaborn.pydata.org [88]).


Model-based approaches to data generation can be ideally based on the knowledge of the data generation process. For example, the data set shown in Figs 1 and 3 was originally generated using the following recipe:

\begin{align*} {\Phi}_i&=U\left(0,2\pi \right) \\ \cos \left({\theta}_i\right)&=\left\{\begin{array}{c}\!\!\!\!\!\!\!\!\!\!\!\!\!\!\! U\left(-\mathrm{0.1,0.1}\right)\ for\ Cls=1\\{}\!\!\!\! U\left(-1,1\right)\ for\ Cls=\left[2,\dots, 7\right]\end{array}\!\!\!\!\right\}\\ \sin \left({\theta}_i\right)&=\sqrt{\left(1-\cos \left({\theta}_i\right)\right)\bullet \left(1+\cos \left({\theta}_i\right)\right)} \\ radius&=r\bullet U{\left(0,1\right)}^{\frac{1}{3}} \\ Var1:\mathrm{x}&= radius\bullet \sin \left({\theta}_i\right)\bullet \mathit{\cos}\left({\Phi}_i\right)+{x}_{0,{Cls}_k} \\ Var2:y&= radius\bullet \sin \left({\theta}_i\right)\bullet \mathit{\sin}\left({\Phi}_i\right)+{y}_{0,{Cls}_k} \\ Var3:z&= radius\bullet \cos \left({\theta}_i\right)+{z}_{0,{Cls}_k} \end{align*}

with the data cloud centers located at


(1) \begin{equation*} {\displaystyle \begin{array}{c} Cls\\{}1\\{}2\\{}3\\{}4\\{}5\\{}6\\{}7\end{array}}\left[\begin{array}{ccc}{x}_0& {y}_0& {z}_0\\{}0& 0& 0\\{}3& 0& 0\\{}-3& 0& 0\\{}0& 3& 0\\{}0& -3& 0\\{}0& 0& 3\\{}0& 0& -3\end{array}\right] \end{equation*}

A total of $n=212$ data points are drawn from these equally from the $k=7$classes, $Cls$. The data clouds are constructed around seven centers on the main axes of a 3D coordinate system (Fig. 1A). For all classes except the origin (${x}_0,{y}_0,{z}_0=\left[0,0,0\right]$), $n=30$ points are randomly drawn around each center within a sphere of radius $r=1$. For the data points centered at the origin, $n=32$ points are randomly drawn within a sphere of radius $r=0.1$, so that this data cloud has 10 times the data density of the other six data clouds. The data set is named “Hepta” and is part of a collection of canonical datasets called “Fundamental Clustering and Projection Suite” (FCPS [25]).

When the analytical solution for a given data set is not available, as is often the case for complex biomedical datasets, a probabilistic approach is commonly used [7]. This means that the given data are a finite example of a known and mathematically defined probabilistic structure. In particular, the structure of all the distributions used for the generation can be mathematically formulated and an observed data set is assumed to be a finite sample from a potentially infinite data set that follows some parametric or nonparametric distribution [26, 27]. The underlying distribution is then modeled, based on implicit assumptions or prior knowledge about how the data are expected to be distributed. This approach assumes that the distribution of the data set is known and agrees with these implicit assumptions. However, this assumption may not always hold, especially for biomedical and other real-world datasets, where the true underlying interrelations are often complex and not well characterized [28, 29]. Studies have shown that many biological datasets exhibit nonstandard distributional properties, such as heavy tails, multimodality, or other deviations from common probabilistic models like the normal distribution [30, 31]. This lack of knowledge about the true data-generating distributions is a key limitation of probabilistic model–based generative approaches that rely on assumed parametric or nonparametric distributions [7].

### Leveraging emergent self-organizing maps for data structure detection and generation

The emergent SOM (ESOM)–based method proposed here aims to circumvent this issue by learning the intrinsic data structures directly from the observed samples, without making probabilistic assumptions about the underlying distribution. We propose the use of unsupervised learned neural networks, specifically SOMs, for a model-free representation, however, neighborhood (topology) preserving of empirical data. These unsupervised neural networks can capture and represent the intrinsic structures of multivariate data spaces through their learning process, without relying on any a priori knowledge.

#### Brief overview of emergent self-organizing maps

ESOMs are an advanced class of topographic maps that provide a method for visualizing and exploring complex data structures. Developed from Kohonen’s SOMs [22], ESOMs create a disentangling projection of high-dimensional data onto a 2D grid, preserving the topology and neighborhood structure of the original dataset [23]. This unsupervised machine learning technique uses a network of neurons arranged on a 2D grid, where each neuron’s weight vector represents a point in the high-dimensional space. Through an iterative learning process inspired by biological brain structures, ESOMs adapt these weight vectors based on similarity to presented data points, gradually refining the projection. An atomic learning step of the SOM consists of drawing the weight vector of the best match and, to a lesser extent, the weight vectors of its neighbors toward the presented case. As the learning neighborhood decreases over time, the resulting map effectively preserves topological relationships and disentangles intricate data structures, making ESOMs particularly valuable for visualizing and analyzing complex, high-dimensional data sets [25].

ESOMs facilitate the detection of neighborhood structures and voids in the data space [22] using the U-matrix [32]. Constructed on a 2D grid of neurons, the U-matrix assigns a height to each neuron based on the average distances to its nine neighboring neurons, where large U-heights indicate distant data points (voids) and low U-heights represent dense data regions. This topographic representation reveals the high-dimensional data structure as hills or ridges separating valleys filled with similar data clusters. Complementing the U-matrix, the P-matrix measures the data density at the location of each neuron, further enhancing the visualization. Consequently, a structured dataset will show valleys in the U-matrix corresponding to dense data clusters, with high densities in the P-matrix separated by hills in the U-matix and lows in P-matrix indicating voids in the data space.

#### Emergent self-organizing map–based density estimation for multivariate data

The ESOM has been demonstrated in many applications that such neural networks can effectively capture and process the features of high-dimensional data, such as in transcriptomic analyses of normal human tissues and tumor samples [33]. After the self-organizing learning process, the ESOM represents the high-dimensional data space on a 2D grid of neurons. The U-matrix and P-matrix add a third dimension to this representation. The U-matrix heights above a neuron $n$ within the 2D grid define the average distances between the data represented in the Moore neighborhoods of neuron $n$ [34]. The P-matrix captures the density of the data space at the location of the data point represented by the weight vector of neuron $n$ [23]. This 3D representation visualizes the distance and density structure of the data. Regions with dense data clouds and small average distances appear as valleys, while empty spaces with large distances and low density appear as hills on the U-matrix and ditches on the P-matrix. This allows assessing whether the multivariate data are structured in dense data clouds with small average distances or if there are empty space regions with large distances and low density.

**Figure 4:**
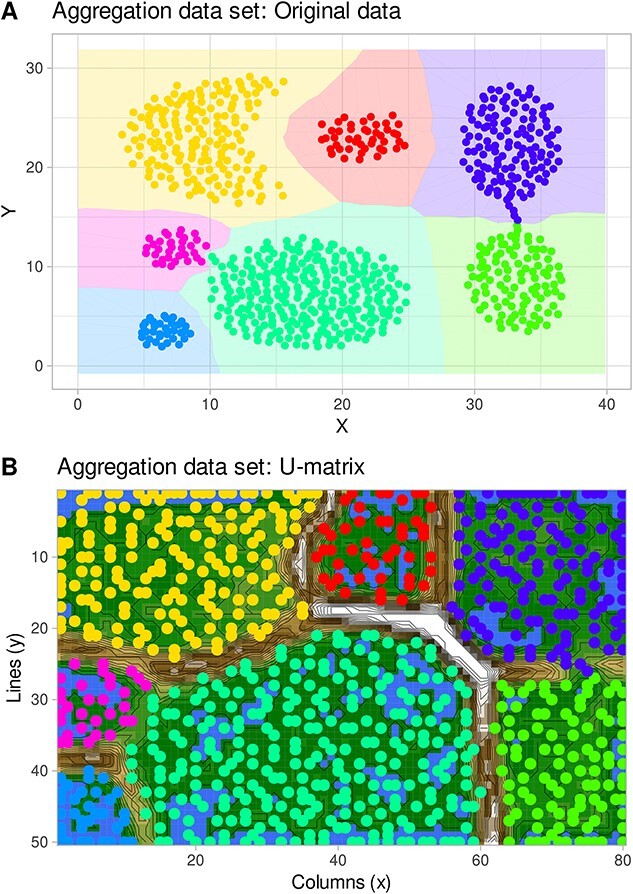
“Aggregation” dataset (http://cs.joensuu.fi/sipu/datasets/). (A) 2D representation of the original dataset, with individual instances color-coded by class on the background of a class-wise Voronoi tessellation. (B) ESOM projection of the dataset. The classes are separated by high “snow-covered” ridges. The figure has been created using the R software package (version 4.3.3 for Linux; https://CRAN.R-project.org/ [89]) and the R libraries “ggplot2” (https://cran.r-project.org/package=ggplot2 [90]).

Probability density estimation for multivariate data sets is a difficult task [35]. For big data, where performance issues are critical, kernel density estimation using hyperspheres (spheres) with a global radius is efficient and performant [36]. A sufficiently trained ESOM projects the high-dimensional $d$ data points $\left\{{x}_i,i=1\dots n\right\}\ in\ X\subseteq{R}^d$ neighborhood preserving onto a 2D grid $\left[1:l\right]\times \left[1:c\right]\subset{N}^2$ of $m=l\cdotp c$ neurons, respectively, their associated weight vectors $\left\{{w}_1,\dots, {w}_m\right\}=W\subset{R}^d$. The projections of the points *x_i_* are the corresponding weight vectors $\left[w\left({x}_i\right)\right]$. If the size of the output mesh is sufficiently large, it is called ESOM [34]. The weight vectors of the ESOM reside in the data space and interpolate and extrapolate with neighborhood-preserving properties. Correctly constructed ESOM should preserve the topology of the input data (Fig. 4).

The P-matrix displays local densities, estimated as the number of data points in a hypersphere of radius $r$ around each weight vector (${w}_i$) of a neuron on the output grid of the ESOM.


**Figure 5:**
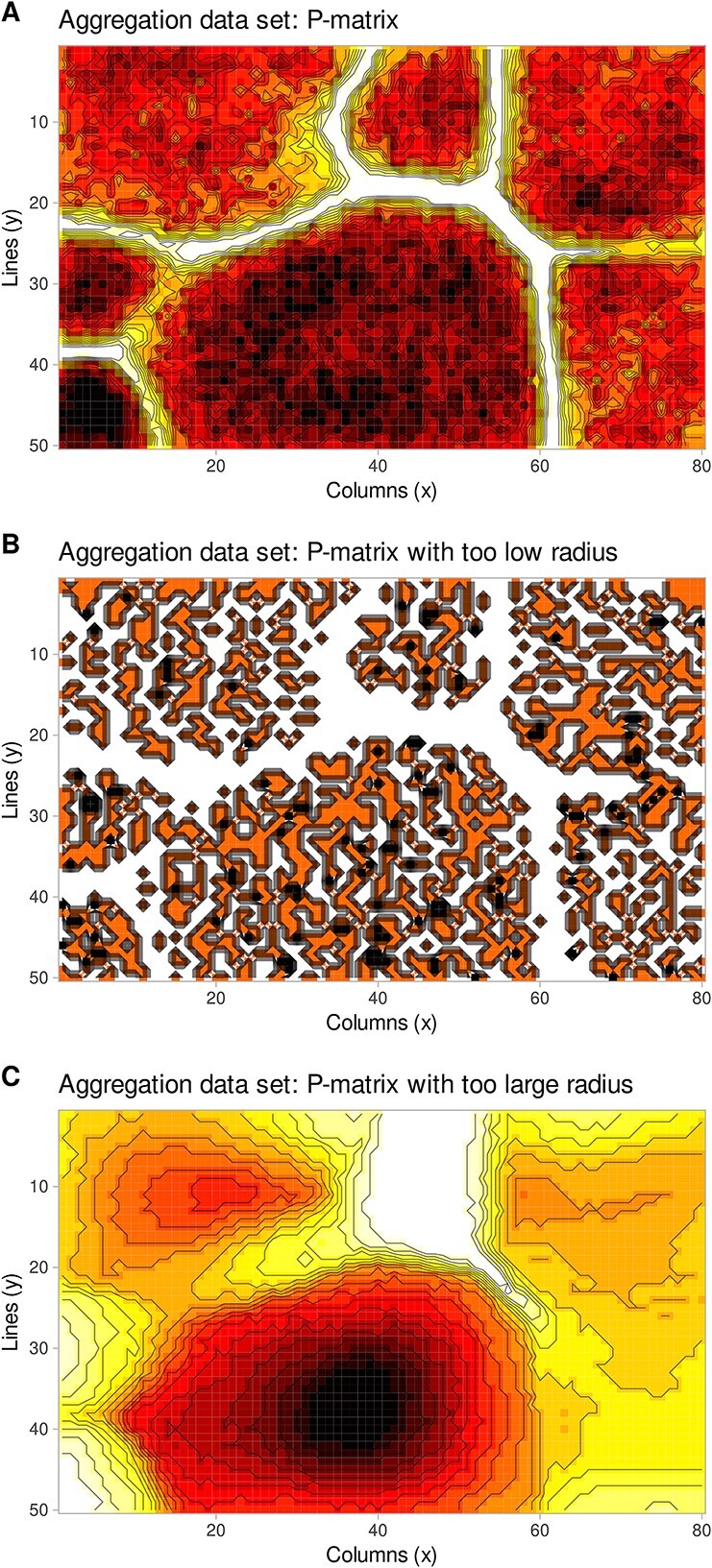
Effect of the length of the radius *r* of a hypersphere around each weight vector of a neuron on the output grid of the ESOM for the estimation of the local data density. (A) Appropriate radius. (B) Too-small radius. (C) Too-large radius. Compare Fig. 4. The figure has been created using the R software package (version 4.3.3 for Linux; https://CRAN.R-project.org/ [89]) and the R libraries “ggplot2” (https://cran.r-project.org/package=ggplot2 [90]) and “ggforce” (https://cran.r-project.org/package=ggforce [91]).

(2) \begin{equation*} Pheight\left({n}_{i,j}\right)=\left|\left\{x|d\left({w}_{i,i}\le r\right)\right\}\right| \end{equation*}

where ${n}_{i,j}$ is the neuron of the U-matrix at row $i$ and column $j$ with weight vector ${w}_{i,i}$. Given these propositions of a correctly constructed ESOM, the P-matrix provides a 2D representation of the multivariate density function of the data space, which is particularly good for density-based separation of different classes (clouds) in the data, including the possibility of estimating whether the radius $r$ is suitable for density estimation. Figure 5B shows the effects of a too-small radius $r$ for density estimation: many zero-valued P-heights and many small pumps in the densities. Conversely, a too-large radius $r$ inflates the intercloud space since points from other classes are included. This can be detected in comparison of the P-matrix to the U-matrix. If large U-heights are located at large densities, the radius $r$ is too large (Fig. 5C). This is due to the inclusion of data points from different data clouds in the hyperspheres (crosstalk phenomenon).

**Figure 6:**
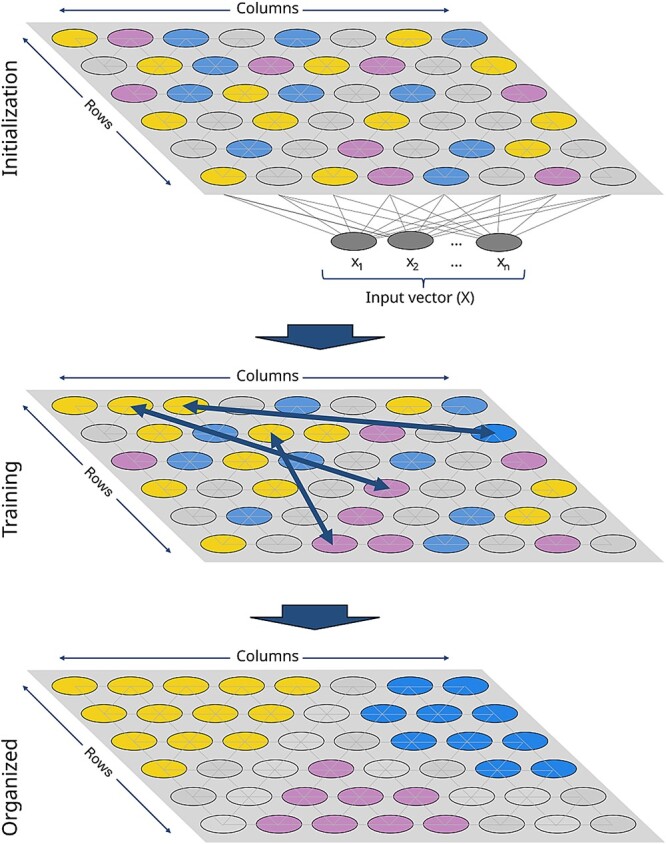
A schematic drawing of an SOM illustrates how input data are initially assigned randomly to neurons. During the training phase, data points adjust their positions, allowing similar data points to cluster in distinct regions. This process continues until similar data points are projected in proximity on the projection plane, occupying specific neurons on the grid, which are referred to as BMUs. The input layer is omitted in the lower two drawings. The figure was created using the free and open-source vector graphics editor Inkscape (version 1.2.2 for Linux; http://www.inkscape.org/).

The optimal parameters for ESOM have been studied previously, leading to several key recommendations [37]. One of the most important parameters is the grid size. As elaborated previously [38], SOMs have two prototypical uses for neurons: representing clusters and projecting high-dimensional data. In the first type, a small number of neurons correspond to data clusters, which works similarly to *k*-means clustering [39]. The second type, which is used in this report, uses a large number of neurons, often thousands, to map and reveal intrinsic structural features of high-dimensional data, a phenomenon known as ESOMs [23] (Fig. 6). The number of neurons and the shape of the projection grid are chosen to balance three important criteria: (i) it should be large enough to avoid degenerating into a *k*-means clustering algorithm [39], (ii) it should be small enough to prevent individual data points from occupying separate neurons with surrounding interpolation regions, and (iii) it should have edge ratios between 1.2 and 1.6 for optimal performance [37]. A starting point of 4000 neurons (80 × 50) has been effective in many applications, with adjustments made for larger or smaller datasets; however, a rectangular grid of at least 600 neurons, typically arranged in a 23 × 30 configuration, seems to mark a minimum [32]. Increasing the grid size increases the resolution of structural features of the data in the resulting visualization. In contrast to the number of neurons, the shape of the neighborhood and the cooling scheme, which refers to the reduction of the neighborhood during learning, were found to be less critical factors [32]. For practical applications, it is often sufficient to use default settings from published packages [40]. The effectiveness of the chosen parameters can be evaluated by examining the resulting U- and P-matrix visualizations, which provide insights into the structure and density distribution of the data.

#### Emergent self-organizing map neural network–based model-free data generation

**Figure 7:**
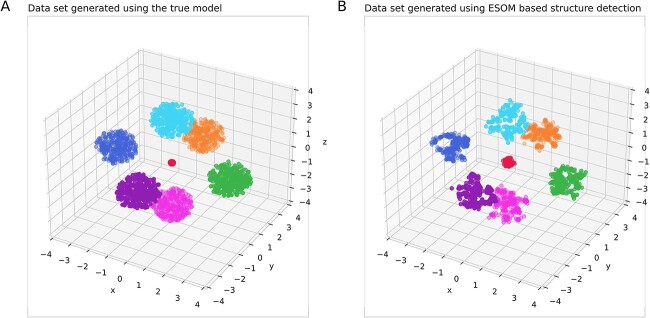
Augmentation of a small data set using either the true data-generating probabilistic model (A) or ESOM-based generative AI (B). The original synthetic “Hepta” dataset consists of *n* = 212 data points evenly distributed across *k* = 7 classes within well-defined and well-separated spherical data clouds (Fig. 1A). The data were generated with 10 times the sample size of the original. The variant shown in (B) was obtained using data generation [Equation (1)]. In contrast, the variant shown in (B) was generated using the proposed ESOM structure detection–based generative AI. The figure was generated using Python version 3.11.5 for Linux (https://www.python.org) with the seaborn statistical data visualization (https://seaborn.pydata.org [88]).

In this report, a generative use of ESOM is proposed as a novel addition to the established use of ESOM as a valid structure recognition type of AI. As the result of an introductory experiment, Fig. 3 shows two augmented data sets from the example above. Both contain 10 times more data than the original data set, where one variant was obtained using the true data generation probabilistic model [Equation (1)], while the other variant was generated using the ESOM-based structure detection with subsequent generation of new data points (Fig. 7). Both new data sets contained only cases that were not identical to any case in the original data set. For trained random forests (RFs) [41, 42] or support vector machine (SVM) [43] classifiers, it was not possible to distinguish the data generated by the true probabilistic model from the data generated by the ESOM-based generative algorithm proposed here (Table 1). The details of this experiment are given later in this report.

**Table 1:** Balanced accuracy of random forest classifiers trained with the task to either detect whether a dataset instance is original or generated (classification task: “Generated data”) or to distinguish between the original classes of the dataset (classification task: “Class assignment”).

Data set	Classification task								*P*-value range generated versus original
	Generated data				Class assignment				
	Original		Permuted		Original		Permuted		
	RF	SVM	RF	SVM	RF	SVM	RF	SVM	
Aggregation	0.5 (0.5–0.5)	0.48 (0.46–0.536)	0.5 (0.5–0.5)	0.498 (0.472–0.527)	1 (0.998–1)	1 (0.998–1)	0.5 (0.5–0.501)	0.5 (0.5–0.511)	0.99–1
FCPS: Target	0.495 (0.491–0.506)	0.499 (0.499–0.5)	0.499 (0.493–0.514)	0.5 (0.499–0.509)	0.998 (0.985–1)	1 (1–1)	0.505 (0.475–0.561)	0.553 (0.403–0.597)	0.93–1
FCPS: Chainlink	0.495 (0.491–0.503)	0.51 (0.486–0.538)	0.499 (0.498–0.505)	0.5 (0.488–0.569)	0.97 (0.958–0.992)	1 (1–1)	0.502 (0.305–0.738)	0.47 (0.247–0.689)	0.87–1
FCPS: WingNut	0.503 (0.494–0.519)	0.489 (0.46–0.513)	0.497 (0.493–0.51)	0.5 (0.472–0.538)	0.999 (0.999–1)	0.999 (0.999–1)	0.5 (0.176–0.805)	0.5 (0.006–0.999)	0.96–0.97
FCPS: Atom	0.472 (0.466–0.48)	0.499 (0.496–0.503)	0.499 (0.497–0.506)	0.5 (0.485–0.527)	0.982 (0.972–0.99)	1 (1–1)	0.469 (0.217–0.724)	0.51 (0.208–0.812)	1–1
FCPS: EngyTime	0.496 (0.49–0.506)	0.499 (0.497–0.5)	0.498 (0.492–0.518)	0.5 (0.497–0.503)	0.951 (0.943–0.958)	0.96 (0.951–0.964)	0.502 (0.163–0.845)	0.5 (0.338–0.712)	0.97–0.97
FCPS: GolfBall	0.5 (0.497–0.512)	0.5 (0.5–0.5)	0.499 (0.497–0.506)	0.5 (0.5–0.5)	–	–	–	–	0.95–0.99
FCPS: Hepta	0.49 (0.484–0.496)	0.501 (0.462–0.537)	0.5 (0.494–0.51)	0.494 (0.461–0.543)	1 (1–1)	1 (1–1)	0.502 (0.425–0.574)	0.5 (0.465–0.567)	0.93–1
FCPS: Tetra	0.491 (0.488–0.495)	0.513 (0.495–0.537)	0.499 (0.497–0.505)	0.508 (0.483–0.545)	0.997 (0.992–0.998)	0.999 (0.998–1)	0.515 (0.369–0.669)	0.499 (0.437–0.546)	0.97–0.98
FCPS: TwoDiamonds	0.502 (0.492–0.515)	0.504 (0.486–0.521)	0.498 (0.492–0.511)	0.502 (0.475–0.524)	0.999 (0.997–1)	0.999 (0.998–1)	0.5 (0.146–0.777)	0.5 (0–0.999)	0.86–0.96
FCPS: Lsun	0.495 (0.49–0.503)	0.5 (0.5–0.5)	0.499 (0.493–0.512)	0.5 (0.5–0.5)	1 (1–1)	1 (1–1)	0.5 (0.5–0.5)	0.5 (0.5–0.507)	0.98–0.99
Leukemia	0.5 (0.5–0.5)	0.454 (0.444–0.464)	0.5 (0.5–0.5)	0.5 (0.5–0.5)	0.996 (0.988–0.999)	1 (1–1)	0.5 (0.5–0.5)	0.5 (0.5–0.5)	0.98–1
PsA	0.5 (0.5–0.5)	0.5 (0.5–0.5)	0.5 (0.5–0.5)	0.5 (0.47–0.53)	1 (0.82–1)	1 (1–1)	0.5 (0.5–0.5)	0.5 (0.43–0.76)	0.99–1

Training was performed with the original variables and again with all variables randomly permuted. Shown are medians and nonparametric 95% confidence intervals (2.5th–97.5th percentiles) of the balanced accuracies from 100-fold cross-validation runs, with training on 80% of the data and validation on the remaining 20% not seen during training. The last column shows the *P*-value range of Mann–Whitney U tests of original versus generated data for all variables in the data set. RF, random forest; SVM, support vector machine.

##### Definition of criteria for a valid generation of data

As a result of the above basic considerations, the main goals of the ESOM-based data generation approach are (i) to preserve existing data structures and (ii) to avoid the introduction of spurious structures. To achieve these goals, the following steps are crucial: first, the data structures must be identified. This includes identifying the regions of empty space and the data clouds within the original multivariate data set and analyzing the data clouds’ characteristics, such as their shape, size, and density. Second, the structural, i.e. topological, properties of the data set should be preserved, which can be achieved by ensuring that any newly generated data are positioned within the existing data clouds while preserving the observed structural properties of the original data, such as the shape, size, and density of the data clouds. The result can be validated by comparing the structural features of the generated data with those of the original multivariate data set, which ideally should be indistinguishable. By focusing on these key steps, the ESOM-based approach aims to generate additional data that are structurally equivalent to the original dataset, without introducing false or misleading structures. This is a critical aspect of the method, as preserving the intrinsic data structures is essential to maintaining the validity and representativeness of the augmented dataset. Generating new data that match the observed characteristics of the original data helps to avoid biasing any subsequent use of the extended dataset.

The goals of data generation are illustrated in Fig. 3. The desired valid data generation provides a data set with respect to data clouds and empty space that follows the original data set (Fig. 3A and B). It does not alter the original structure, such as making the boundaries between data clouds indistinct (Fig. 3C), nor does it introduce new structures, such as additional classes that were not present in the original data set (Fig. 3D). It is worth noting that the generation of additional data with the same structure of a given data set can and should include points that lie within the data clouds as well as in the voids. However, care must be taken not to introduce spurious structures that were not present in the empirical data. The ESOM method described here uses distance (U-matrix) and density (P-matrix) measurements within the empirical data to ensure this.

##### Emergent self-organizing map neural net–based generative algorithm

Generating data points from given data seems like an easy task: to generate new data for each data point (“seed”), simply add a small amount in any direction (“jitter”). However, for multivariate data, this leads to the “curse of dimensionality” [44]. For a single data dimension, the distances of data drawn from a Gaussian distribution follow a distribution with many small and few large distances. However, if the dimensionality of the data is large, the distances generated by such a random process are mainly concentrated around a specific mean. More annoyingly, the difference between the maximum distance and the minimum distance of the generated data (the range) goes to zero [45]. This means that the naive generation of data in the multivariate case has the unwanted effect that the generated data lies on the surface layer of a hypersphere around the seed. As the dimension of the data increases, the radius of the hypersphere increases and the thickness of the layer decreases. For typical empirical data with structure, a second problem arises: the generated data should not blur the boundaries of the neighborhoods or generate data from a seed in one neighborhood that resides in other neighborhoods beyond the empty spaces.

To avoid these effects, a data point’s neighborhood is defined in terms of the distance to the other points. The neighborhood of datapoint ${x}_i$ is described as the probability that another datapoint ${x}_j$, with distance $d\left({x}_i,{x}_j\right)$ from ${x}_i$, belongs to the neighborhood of datapoint ${x}_i$. Mathematically, the probability ${p}_{ij}$ can be formulated as


(3) \begin{equation*} {p}_{ij}=1-\frac{1}{1+{e}^{-\frac{10\bullet d\left({x}_i,{x}_j\right)}{c-1}}} \end{equation*}

where ${p}_{ij}$ is the probability that data point${x}_j$ is in the neighborhood of data point ${x}_i$, $d\left({x}_i,{x}_j\right)$ is the distance between data points ${x}_i$ and ${x}_j$, and $c$ is a scaling constant that determines the size of the neighborhood. This equation represents a sigmoid-like function that maps the distance between two data points to a neighborhood probability. The key aspects are that the function first takes the distance $d\left({x}_i,{x}_j\right)$ and scales it by the constant $c$ to determine the relative distance. The called distance is then qualified by the logistic distribution function. This type of neighborhood probability function is commonly used in SOMs and other unsupervised learning algorithms to capture the local structure of the data. By defining neighborhoods in this way, the ESOM approach can effectively represent the intrinsic data structures, as described in the previous text.

The factor $c$ scales the data’s distances such that the decision boundary is at, i.e. ${p}_{ij}=0.5\ for\ \frac{d\left({x}_i,{x}_j\right)}{c}=1$. This represents the point where a Bayes decision changes from “${x}_j$ is in the neighborhood of ${x}_i$” to “${x}_j$ is not a neighbor of ${x}_i$.” The algorithm generates $k$ points for each data point by first selecting $k$ randomly distributed points on a unit hyper ball around the seed. Then, the length of the distances of these $k$ points to the seed is extended to lengths according to the neighborhood probabilities ${p}_{ij}$ using three different uniform distributions: a large portion containing $85\%\bullet k$ of the generated data is uniformly drawn from distances with ${p}_{ij}>95\%$. Fifteen percent of the generated data are drawn uniformly for distances with $10\%<{p}_{ij}<95\%$ and the remaining $5\%\bullet k$ having distances with $\frac{d\left({x}_i,{x}_j\right)}{c}\le 2$. This algorithm captures the “neighborhood and empty space” structures of the data set such that most of the data are within the neighborhood and only a few data are in the empty space regions. If the P-matrix shows a good choice of $r$ (see Fig. 6A–C), then this represents a distance that can separate the neighborhoods in the data. Factor $c$ is then calculated so that a data point near the center between the two neighborhoods measures “empty space.” This means that $c=0.4\cdotp r$ is a good choice for the calculation of ${p}_{ij}$.

Thus, for multivariate density estimation, the above-mentioned radius $r$ is the critical parameter. Using ESOM, a suitable radius $r$ can be found as follows: the abstract U-matrix heights ($AU\_ heights$) [46] are the subsets of data distances


**Figure 8:**
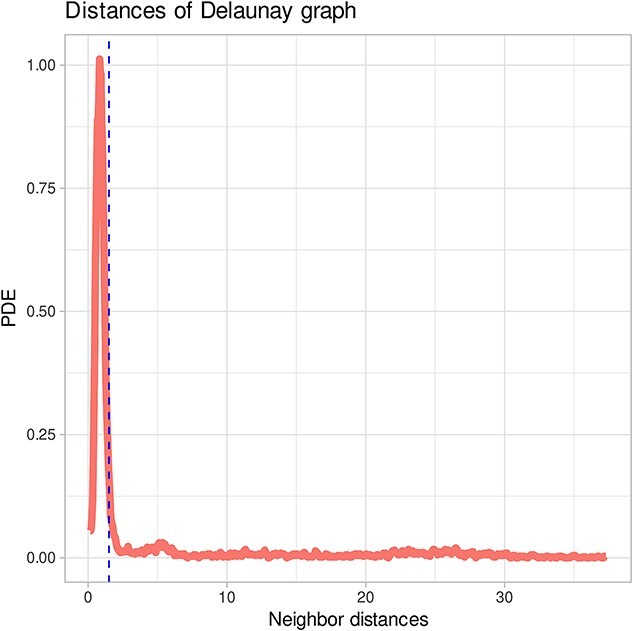
Selection of the radius of the hyperspheres for density estimation based on a proposal obtained from the probability density distribution of the distances between data points. The figure has been created using the R software package (version 4.3.3 for Linux; https://CRAN.R-project.org/ [89]) and the R library “ggplot2” (https://cran.r-project.org/package=ggplot2 [90]).

(4) \begin{align*} \left\{A{U}_{heights}\right\}\subseteq D=&\left\{{d}_i|{d}_i=\mathrm{d}\left({\mathrm{x}}_j,{\mathrm{x}}_k\right)\mathrm{for}\ \mathrm{all}\ \mathrm{pairs}\ \left(\mathrm{j},\mathrm{k}\right)\ in\ G,\mathrm{j},\right.\nonumber\\&\left.\mathrm{k}\in \left[1,\dots, \mathrm{n}\right]\ \right\} \end{align*}

corresponding to edges in the Gabriel graph G constructed from the best-matching units (BMUs). The univariate distribution of AU-heights is represented with a bimodal Gaussian mixture probabilistic model (GMM) optimized for maximum likelihood [expectation maximization (EM) algorithm; Fig. 8]. Using Bayes’ theorem, posterior probabilities can be computed for small distances $p\left( small|d\in D\right)$ in data space, i.e. presumably inner-class distances, and for large distances $p\left( large|d\in D\right)$. A value of $r=0.8\cdotp{t}_{AU}$, where ${t}_{AU}$ is the Bayesian decision boundary between small and large distances, was found to be appropriate for many practical applications.

### Evaluation

#### Code implementation

The R code shown in Textbox 1 generates new data with the same structure as the input data. The displayed R function has become part of the R package “Umatrix” since its version 4.0 is freely available at https://cran.r-project.org/package=Umatrix. The generative process requires the training of the U and P matrices as described previously for this R package [40]. The generative process involves two additional steps. First, the radius *r* [Equation (2)] critical for data generation is obtained via the function “calculate_Delauny_radius(Data, BestMatches, Columns = 80, Lines = 50, Toroid = TRUE),” which expects the data as used for U- and P-matrix generation, the size of the grid in the number of neurons in *x*- and *y*-direction, whether the grid is a toroid, which is the default, and crucially, the best matches obtained as output of the U-matrix generation in this R package. That is, the interactive generation of the U-matrix can be invoked via “<UmatrixName> <- iEsomTrain(Data, Toroid = TRUE, Cls = <priorClasses>”). The best matches are then accessible in the output of the U-matrix generation as <UmatrixName>$BestMatches. Second, the generative process is started by calling the function in Textbox 1, i.e. “generate_data(Data, density_radius, Cls = NULL, gen_per_data = 10)” that expected as input the matrix of data as submitted to U-matrix generation, the above-mentioned density_radius, the class information of the data as a (numerical) vector, and the number of new instances per original instance to be generated (gen_per_data). The data generation process employs a density radius parameter, multiplied by a constant of 0.4 (line 16 in the code in Textbox 1), to create new data points for neighboring classes. This approach ensures that generated points for one class do not encroach upon the data range of adjacent classes, maintaining class structure integrity. Extensive testing has shown that using a factor slightly below 0.5 (which would place new points exactly halfway between classes) yields optimal results. Setting the factor too low would unnecessarily limit the variance of generated points, producing data too similar to the original set, i.e. not making use of the variation allowed within the detected data set structure. Conversely, a higher factor risks blurring class boundaries. This method aims to fill the space around each data cloud as much as possible without invading neighboring clouds, effectively populating the empty spaces between dense data point clusters in the initial data space partition.

**Textbox 1:** R code to generate new data points, given the P-matrix-based radius of the hypersphere around each weight vector of a neuron on the ESOM output grid, which provides an estimate of the local data density.

# Function to generate new data with the same structure as the input data generate_data <− function(Data, density_radius, Cls = NULL, gen_per_data = 10) { ## Initialize parameters # Maximum distance for data generation (relative to density_radius) max_distance <− 2.0 # Limits for the sigmoid function limit_ab <− 0.72 limit_bc < − 1.22 # Fraction of data in each set (A, B, C) percent_a < − 0.80 percent_b < − 0.15 # Radius for data generation (shrink to below half of the intercloud distance) r < − 0.4 * density_radius # Number of data points and dimensionality n < − nrow(Data) d < − ncol(Data) ## Assign class labels if not provided if (is.null(Cls)) { Cls < − rep(1, n) } else if (length(Cls)! = n) { stop("Unequal number of cases and class labels.") } ## Generate new data points # Number of generated data points n_generated <− n * gen_per_data # Generate jitter (noise) for the data points jitter <− matrix(rnorm(n_generated * d, mean = 0, sd = 1.5), n_generated, d) * r jitter_lengths <− sqrt(rowSums(jitter^2)) jitter_indices <− which(jitter_lengths >0) jitter[jitter_indices,] < − jitter[jitter_indices,] / jitter_lengths[jitter_indices] # Determine the number of data points in each set (A, B, C) n_a < − round(n_generated * percent_a) n_b < − round(n_generated * percent_b) n_c < − n_generated—n_a—n_b # Generate the sigmoid lengths for each set sigmoid_lengths <− c( runif(n_a, 0, limit_ab), runif(n_b, limit_ab, limit_bc), runif(n_c, limit_bc, max_distance) ) * r # Create the sigmoid matrix and generate the new data sigmoid_matrix <− matrix(rep(sigmoid_lengths, d), ncol = d) generated_data <− Data[rep(1:n, gen_per_data),] + jitter * sigmoid_matrix generated_classes <− rep(Cls, times = gen_per_data) # Return the original and generated data and classes return(list( original_data = Data, original_classes = Cls, generated_data = generated_data, generated_classes = generated_classes )) }

The code has been incorporated as a new function in version 4.0 of the R library “Umatrix” (https://cran.r-project.org/package=Umatrix).

In the present experiments, the hyperparameter settings of the U-matrix and P-matrix generation were left to the defaults of the program. The choice of grid size has been described above. For other hyperparameters, such as the tilt rate and the neighborhood function, the choice of other settings and details about their functions have been described in detail [47, 48]. Experiments on data generation using the neural network were conducted on artificial and molecular data sets as described below.

#### Data sets

##### Artificial data sets

A collection of datasets created for benchmarking of clustering and data projection algorithms has been published as part of the “Fundamental Clustering Problems Suite (FCPS)” [25]. It is freely available at https://www.mdpi.com/2306-5729/5/1/13/s1 or in the similarly named R library “FCPS” (https://cran.r-project.org/package=FCPS [49]). It has been found that the structures in this dataset are comprehensively captured by the ESOM/U-matrix method as the basis of the present generative algorithms [38]. Details of the data subsets have been described elsewhere [25]. In brief, the FCPS contains 10 data sets with the names “Atom,” “Chainlink,” “EngyTime,” “Golfball,” "Hepta,” “Lsun,” “Target,” “Tetra,” “TwoDiamonds,” and “WingNut.” The data sets consist of $n=212\hbox{--} 4096$ data points. A further synthetic data set was the “aggregation” data set (http://cs.joensuu.fi/sipu/datasets/) that is designed to show how data aggregation can be used to improve the quality and robustness of data cloud detection algorithms.

##### Biomedical data sets

A 100-dimensional biomedical transcriptomics dataset was selected for the inclusion of rare disease settings. The “Leukemia” data set consists of $n=552$ microarray data comprising $d=100$ differentially expressed genes from $n=108$ healthy subjects, $n=266$ patients with acute myeloid leukemia (ALL) and $n=163$ patients with chronic lymphocytic leukemia (CLL) [50]. A small subgroup of patients (*n* = 15, 3%) suffered from APL, which has an incidence of <4% of all leukemia cases in Germany [4].

Another biomedical dataset was available from an ongoing clinical study on the lipidomics background of psoriatic arthritis (“PsA” data set). In a cross-sectional clinical study setting, $n=81$ patients presenting with possible arthritic complications of psoriasis were enrolled consecutively. Lipid markers ($d=293$) of several classes (e.g. acylcarnitines, ceramides, cholesterol esters, diacylglycerols, endocannabinoids, sphingoid bases, triacylglycerols, and others) were analyzed in the patients’ plasma using locally developed liquid chromatography–mass spectrometry assays. Healthy control samples were obtained from a local blood bank, but the donors from whom the samples had been obtained turned out to be younger than the patients. Since many lipid variables showed a significant correlation with age, matching was considered necessary. However, after age matching, only $n=16$ controls were available in the analyzed data set. The study from which these data were used was conducted in accordance with the Declaration of Helsinki on Biomedical Research Involving Human Subjects and was approved by the Ethics Committee of the Medical Faculty of the Goethe University, Frankfurt am Main, Germany (approval number 19-492_5). Informed written consent was obtained from each participant, including for anonymized use of data for assessments such as those reported here.

#### Detecting artificial intelligence–generated content in mixed datasets

In a supervised discriminator experiment, 20% of the data were first separated from the pooled original and generated data set in a class-proportional manner. The 20%-validation subsample was not touched during subsequent classifier training. This approach allows for a robust evaluation of the performance of the machine learning models by testing them on unseen data, which helps to prevent overfitting. Separation of the validation sample was done using the R library “opdisDownsampling” (https://cran.r-project.org/package=opdisDownsampling [51]), which repeats the sampling ${10}^6$ times and compares the distribution of the drawn sample with the distribution of the original data, obtaining subsets of data that better reflect the entire data set than a first randomly selected subsample [51].

Subsequently, machine learning algorithms were trained with the task of detecting which data point was generated and which was original. Specifically, a 100-fold cross-validation scenario was implemented. This setup involved training of classifiers of two different types, i.e. RF [41, 42] as a robust tree-based bagging classifier and SVM [43] as a hyperplane separation–based method, on two-thirds of the aforementioned 80% training/test data subset, followed by validation on 80% of the cases randomly selected from the 20% validation sample not seen by the classifiers during training. Random sampling during each of the 100 runs was done using class-proportional Monte Carlo sampling [52]. The primary measure of success was the balanced accuracy [53] calculated using the R package “caret” (https://cran.r-project.org/package=caret [54]). The classifier training process included initial hyperparameter tuning for each new task and data set, including optimization of the number of trees in the forest and other RF hyperparameters as described in Lötsch and Mayer [55], or selecting the kernel form for SVM from “linear,” “polynomial,” “radial,” or “sigmoid,” and other SVM hyperparameters such as the values for cost or $\gamma$. Random forests were implemented using the R library “randomForest” (https://cran.r-project.org/package=randomForest [56]), while the SVM was used in the implementation of the R package “e1071” (https://cran.r-project.org/package=e1071 [57]). The training process was repeated with the new task of detecting the original classes to ensure that the algorithms were able to classify the datasets; otherwise, a failure to detect the generated data could not be uniquely attributed to the similarity of the generated and original data but could merely indicate that the algorithm did not work at all due to implementation errors. In addition, to control for possible overfitting, the training was replicated with permuted variables (i.e. nonsense information), with the expectation that classifiers trained with them would perform no better than guessing the class assignment, i.e. achieve a balanced accuracy of ~50% with a 95% confidence interval defined by the 2.5th and 97.5th percentiles of the 100 cross-validation runs, spacing the 50% chance level. In addition, all variables of all data sets were compared between their original and generated versions using Wilcoxon–Mann–Whitney U-tests [58, 59].

## Results

### Emergent self-organizing map neural net–based generative algorithm

**Figure 9:**
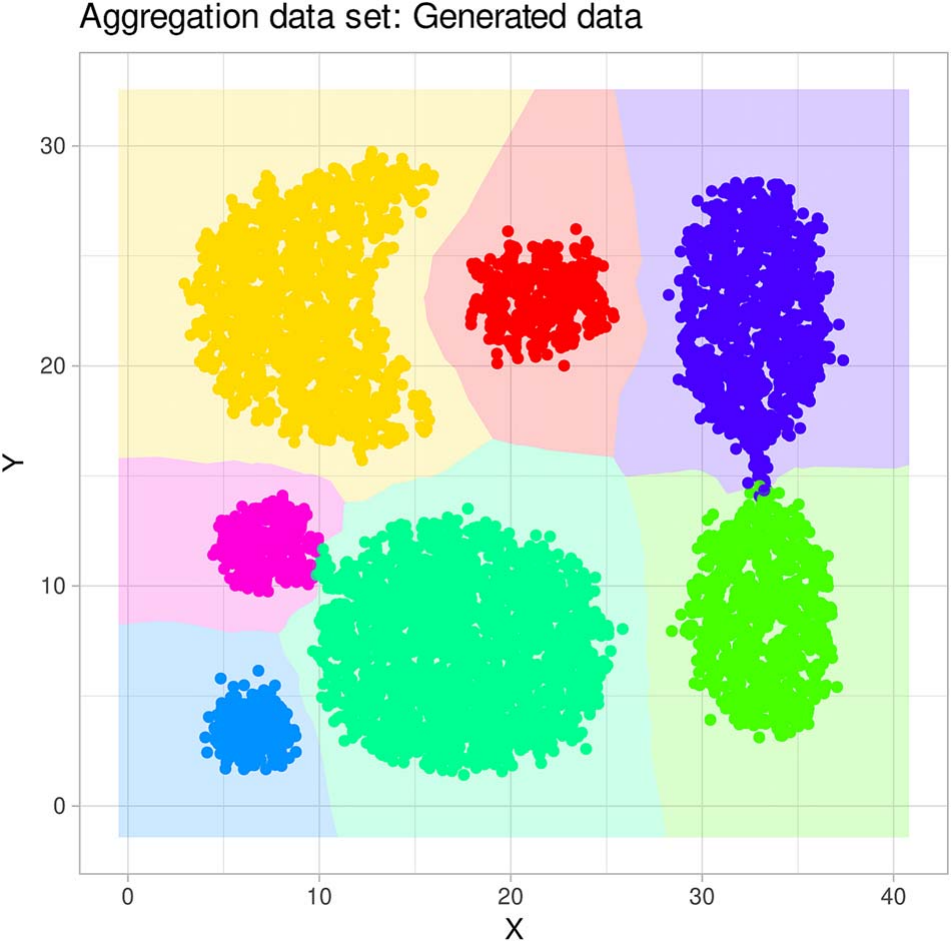
“Aggregation” data set (see Fig. 4) with *k* = 10 newly generated data per original data point. The figure has been created using the R software package (version 4.3.3 for Linux; https://CRAN.R-project.org/ [89]) and the R libraries “ggplot2” (https://cran.r-project.org/package=ggplot2 [90]) and “ggforce” (https://cran.r-project.org/package=ggforce [91]).

The working of the proposed generative algorithm is shown at the “aggregation” data set (http://cs.joensuu.fi/sipu/datasets/). First, a valid ESOM projection of the data set was obtained, i.e. a correctly constructed ESOM should preserve the topology of the input data. Consequently, the projected points of a data cloud are arranged in such a way that data sets located within a data cloud are projected within the representation area of that cloud, and data points that have, possibly, distant neighbors from other data clouds are projected to the edges of these areas (Fig. 4). Within a data cloud, the average distance to neighboring data points on an ESOM is small. This can be seen as low heights on the U-matrix, which shows valleys for the data cloud. Figure 4B shows the U-matrix showing that the seven classes are well separated by high walls, i.e. large local distances. Details of the workflow of data generation including selection of the density radius $r$ are depicted in Fig. 5, showing the impact of selecting a too-small or too-large radius, and in Fig. 8 showing the EM-based selection of the most suitable value of $r$. The result is shown in Fig. 9 depicting a generation of $k=10$ data points for each seed. The “neighborhood and empty space” structures of the original data set are preserved.

**Figure 10:**
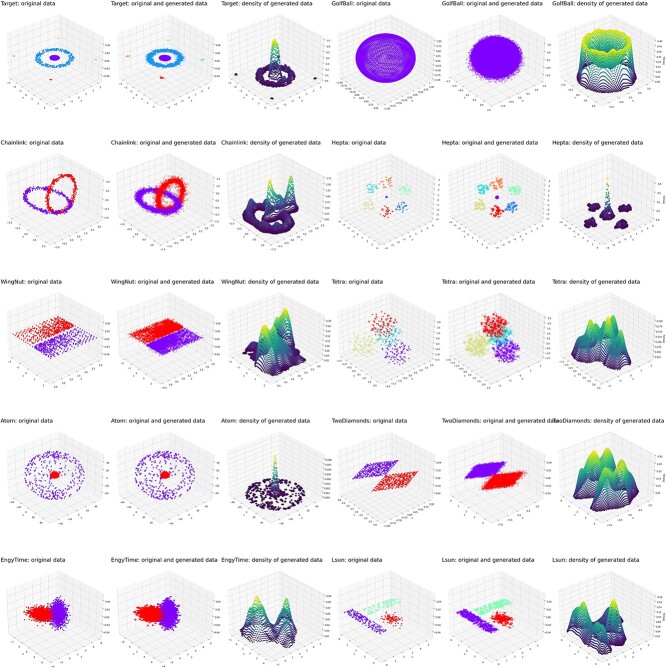
Generation of additional data points in a set of artificial data sets assembled in the so-called FCPS [92]. The FCPS contains a collection of 10 datasets, including “atom,” “Chainlink,” “EngyTime,” “Golfball,” “hepta,” “Lsun,” “target,” “tetra,” “TwoDiamonds,” and “WingNut.” In this figure, each line displays two data sets in three subpanels each. The original data shown on the first panels of a triplet, the added generated data in the middle panels, and the probability of data points in the respective set on the right panels. The figure was generated using Python version 3.8.13 for Linux (https://www.python.org), with the seaborn statistical data visualization package (https://seaborn.pydata.org) used for visualization purposes.

**Figure 11:**
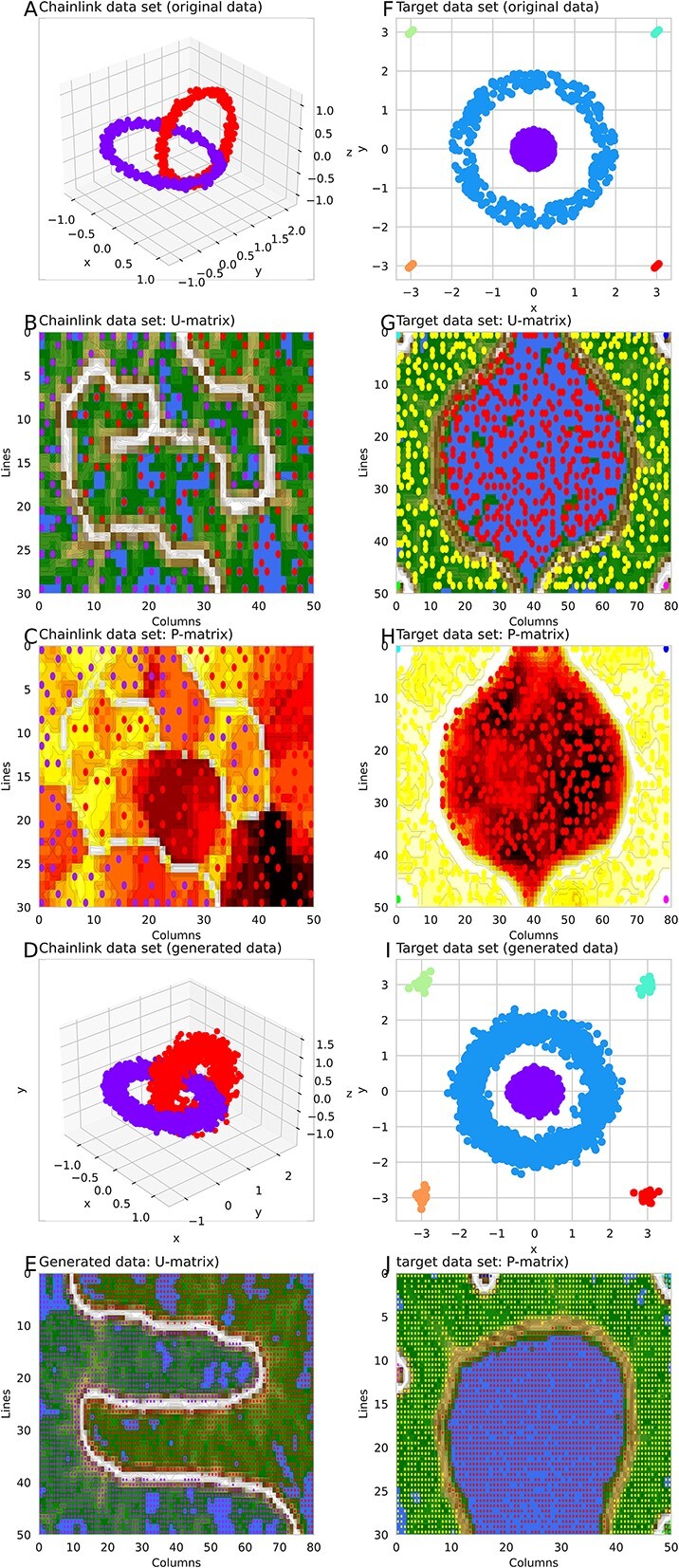
Generation of data in the FCPS “Chainlink” (left column) and “L-sun” (right column) datasets [92]. The top panels (A, F) show the original data. The U-matrix of the data sets (B, G) and the P-matrix (C and H) follow. Generated data are shown in (D) and (I), which when projected onto the SOM result in U-matrix representations (E, J) that show the same subgroup structure as the original data sets (B, G). The figure was created using Python version 3.11.5 for Linux (https://www.python.org) with the seaborn statistical data visualization package (https://seaborn.pydata.org [88]).

Data sets from the “Fundamental Clustering Problems Suite (FCPS)” [25] collection were submitted to the generative algorithm. The full results of structure detection and data generation are shown in Fig. 10, including plots of the original data, the added generated data, and the (similar) distributions of both as 3d density plots. For example, the “Chainlink” dataset contains two distinct classes arranged as interlinked rings. Each class is sampled uniformly from within a torus with minor radius $r=0.1$ and major radius $R=1$. The two tori are orthogonally intertwined with maximum distances between them. This data set was correctly projected by ESOM on the 2D ${R}^2$ plane as two clearly separated classes with a high wall depicted as a “snow-covered mountain range” as a visualization of their separation on the U-matrix (Fig. 11). The ESOM neural net–based generative algorithm added new data points at locations consistent with the original high-dimensional data structure. A new U-matrix created with the generated “Chainlink” data indicated the same class separation as the U-matrix created with the original data set.

### Generated versus original data

In a supervised discriminator experiment, 20% of the data were first separated from the pooled original and generated data set in a class-proportional manner. Using a 100-fold cross-validation design, from the 80% training/test subsample, two-thirds were randomly drawn and used to train an RF classifier. The trained algorithm was then used to solve the task of distinguishing generated from original data in also randomly drawn 80% of the 20% validation sample not used for classifier training. Hyperparameter tuning was performed for each scenario. In all datasets, both RF and SVM classifiers failed to reliably and correctly identify a data instance as either original or generated. In most cases, the median balanced accuracy was close to 50%, marking the guessing level, and, in all cases, the 95% confidence interval of the 100 balanced accuracy values included the 50% guessing level (Table 1). This indicates that, at least for these two well-established classifiers, despite careful hyperparameter tuning, the task of distinguishing generated from original data points was not successfully completed. In fact, the performance of the classifiers was no better than when they were trained with permuted variables, i.e. with nonsense information about class membership. In contrast, the classifiers were very good at learning the classes contained in the datasets for all datasets except the golf ball dataset from the FCPS, which contains no class structures. Balanced accuracy for class assignments often exceeded 90% or better, with the SVM achieving a median balanced accuracy of 100% more often than RFs, which also came close to this ideal. This indicates that the experiments were implemented correctly and that the failure of the classifiers to detect the original data was not due to a general implementation error. Again, training with permuted information resulted in nonfunctional classifiers for the original classes, indicating that the good performance in the above experiment was not an overfitting effect. Furthermore, the statistical comparison of all variables of all datasets between their original and generated versions revealed no significant differences, with the *P*-values of all 419 tests (the total count of all variables in all data sets) ranging from *P* = .86 to *P* = 1. The *P*-value ranges for the individual data sets are shown in the last column of Table 1.

### Augmentation of small data (sub)sets using generative artificial intelligence

#### Synthetic data

The “Target” data set from the FCPS addresses the problem of outliers (Fig. 4). It consists of two large classes with $n=395$ and $n=363$ points on a plane that lie in a circle inside a ring (linearly inseparable). Furthermore, there are four sets of only three points (outliers) that are outside the ring. This data set was correctly projected onto the ${R}^2$ plane, including two clearly separated large classes in the gallop and four small classes at the corners of the projection area, all separated by a high wall represented as a “snow-covered mountain range” (Fig. 11). The SOM neurons that emerged as BMUs during training were colored according to the class labels, indicating that the class separation on the ESOM/U-matrix was perfect. A generative ESOM added new data points at locations consistent with the original high-dimensional data structure. A new U-matrix created with the generated Target data showed the same perfect class separation as the U-matrix created with the original data set. This time, the “outliers” were increased in number and placed on the U-matrix as “volcanic craters” on the physical map analogy as the standard representation of this type of SOM.

#### Biomedical data

**Figure 12:**
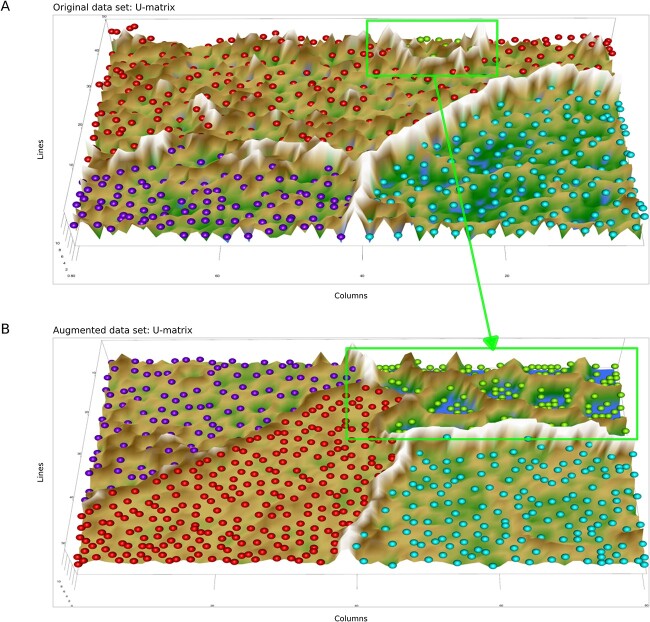
Augmentation of rare cases in biomedical data using generative AI (“Leukemia” data set): (A) 3D representation of the ESOM projection of the leukemia dataset. The subgroups are separated by “snowy mountain ranges.” A small subgroup (green colored best matches at the top/back of the projection surface) contained only 3% of the cases) (marked with a green rectangle). (B) 3D representation of the ESOM projection of the leukemia dataset in which all subgroups have been enlarged by data generation to the size of the original largest subgroup (*n* = 266). The previously small subgroup of APL still appears as a separate subgroup (green best matches) but with a correspondingly larger group size. The figure has been created using the R software package (version 4.1.2 for Linux; https://CRAN.R-project.org/ [89]) and our library “Umatrix” (https://cran.r-project.org/package=Umatrix [40]).

The ESOM works on all dimensions of a data set. A 100-dimensional biomedical dataset was selected for the inclusion of rare disease settings. It consists of $n=552$ microarray data comprising $d=100$ differentially expressed genes from $n=108$ healthy subjects, $n=266$ patients with ALL, and $n=163$ patients with CLL [50]. A small subgroup of patients (*n* = 15, 3%) suffered from APL, which has an incidence of <4% of all leukemia cases in Germany [4]. The classes are well separated by walls (ridges in the U-heights; Fig. 12). Using generative ESOM, an arbitrary number of high-dimensional vector data (gene expressions) can be generated. These vectors consist of the regions of Rn where the measured APL data are located plus the surrounding space. They will have the APL-specific dependency structure between genes. With this larger data set, a fine analysis of the genetic mechanisms of this disease seems more feasible than with only 15 cases. An example is shown in Fig. 12B where all groups have been enlarged to the *n* = 266, which was the size of the largest subgroup. All five data set regions were enhanced to this size, meaning that *n* = 266 − 15 = 251 generated data were added to the APL subgroup, with an analogous addition of *n* = 158, 0, 103 generated cases the other subgroups in the above succession.

**Figure 13:**
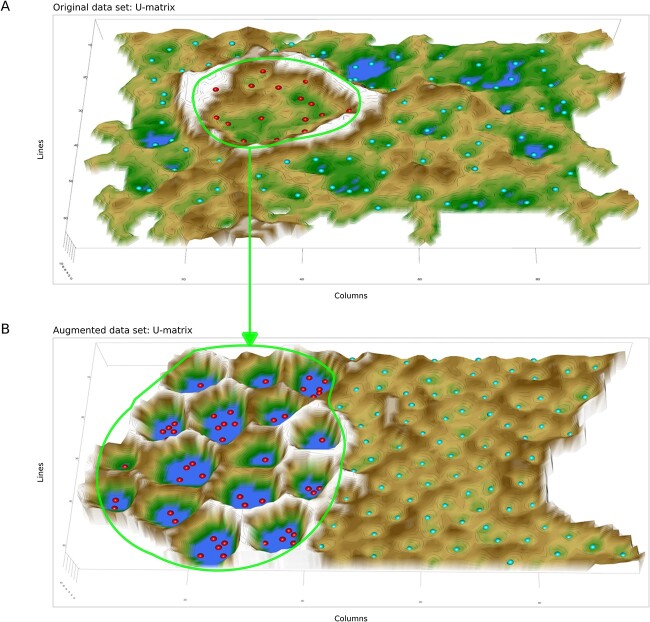
Augmentation of rare cases in biomedical data using generative AI (“PsA” data set): (A) 3D representation of the ESOM projection of the PsA (psoriatic arthritis) dataset. The controls (*n* = 16, red colored best matches) are included in a circular area separated from the PsA patients (*n* = 81) by “snowy mountain range.” (B) 3D representation of the ESOM projection of the PsA dataset in which the two subgroups have been enlarged by data generation to the size of the original largest subgroup (*n* = 81). The previously small subgroup of healthy controls still appears as a separate subgroup (red best matches). The figure has been created using the R software package (version 4.1.2 for Linux; https://CRAN.R-project.org/ [89]) and our library “Umatrix” (https://cran.r-project.org/package=Umatrix [40]).

Similarly, the psoriatic arthritis lipidomics data set (“PsA” data) with a smaller subgroup of *n* = 16 healthy controls was augmented to equal group sizes. The small group of healthy controls was initially separated in a circular region in the middle of the projection area (Fig. 13A). The primary structural feature recognized by the ESOM consists of placing the 16 controls within a “caldera” (valley) surrounded by high “volcano flanks” (green line in Fig. 13A). This indicates that the controls have common properties which are well separated from the 81 PsA patients (green points in in Fig. 13A). After the addition of the generated data, providing *n* = 81 subjects per group, both groups were correctly projected in different regions with no overlap and a main separation ridge along the center of the projection area (Fig. 13B), indicating that the original structure of the data set consisting of two distinct classes (PSA arthritis patients and controls) was preserved with the generative augmentation procedure. The generated data still reside within a large surrounding “caldera” (see the green line in Fig. 13B) indicating that the separation between controls and PsA patients is preserved in the ESOM generated data.

## Discussion

Using the ESOM/U-matrix approach, it is possible to assess whether there is structure in the data or not [38, 40, 60–64]. Thus, the method can correctly detect structures and is also able to correctly detect the absence of a data structure in structureless data [38]. Given this ability of the U-matrix to correctly detect data structures, as discussed above, the method proves highly suitable for basing a data-generating algorithm on. One significant advantage of the ESOM neural net–based generative algorithm is its intuitive visualization of structure, which presents nonlinear representations of high-dimensional distributions and densities in a user-friendly manner. By utilizing the U- and P-matrix, it becomes easy to detect significant structures in the data and to ensure that the data generation parameters are correctly established. Furthermore, identifying outliers and misrepresented data is a simple task. Overall, ESOM-based data generation is a highly desirable option that provides increased ease of use and improved accuracy. Outliers as well as small, underrepresented subgroups of cases of a rare disease, such as APL, can be easily addressed with the present method. Similarly, outliers in clinical data sets, such as patients with rare symptom patterns that can only be mentioned as anecdotal outliers in the raw data set, can easily be included in the systematic analysis up to machine learning–based feature selection, as recently shown for rheumatic diseases using the same method as presented here in full detail [65]. The ESOM U-Matrix method has been applied, in addition to the present leukemia dataset, to various types of omics datasets, including genomics of analgesic drug targets [62], lipidomics data [61, 63], psychophysiological data [66], and many more. The versatility of ESOMs extends far beyond biomedical applications, finding use in diverse fields such as architecture, meteorology, stock market analysis, aeronautics, and even music data clustering [67–69]. This efficient tool for structure recognition is not constrained by data dimensionality or dataset size, though it does require rationally scaled data.

**Figure 14:**
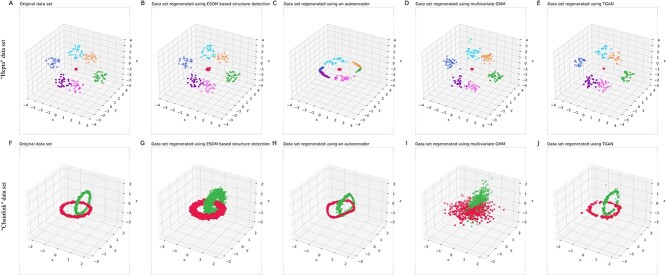
Visualization of reconstruction of the “Hepta” data set [25] comprising *n* = 212 data points evenly distributed across *k* = 7 classes within well-defined and well-separated spherical data clouds (top row) and of the “Chainlink” dataset [25], which consists of *n* = 1000 data points evenly distributed over *k* = 2 classes and arranged in the form of two intertwined rings (bottom row). (A, F) Original data set. (B, G) Similarly sized data set but all data points have been generated using the ESOM neural network–based algorithm. (C, H) Similarly sized data set but all data points have been generated using an autoencoding neuronal network with three hidden layers sized [3, 5]. (D, I) Similarly sized data set but all data points have been generated using a multivariate GMM fit on the original data. (E, J) Similarly sized data set but all data points have been generated using a tabular data synthesizer (TGAN; https://sdv.dev/TGAN/) on the original data. The figure has been created using the R libraries “ANN2” (https://cran.r-project.org/package=ANN2) and “MGMM” (https://cran.r-project.org/package=MGMM [93]), with the final plotting using Python version 3.11.5 for Linux (https://www.python.org) with the seaborn statistical data visualization package (https://seaborn.pydata.org [88]).

The present use of unsupervised neural networks in this report, such as ESOMs, bears similarity to supervised autoencoders in their ability to detect inherent structures within data sets. Akin to how deep learning autoencoders learn to encode and reconstruct input data, our approach utilizes neural networks to identify the underlying structures present in multivariate data. This learned representation then enables the generation of new data points that preserve the detected structural properties of the original data set. With respect to autoencoder, despite the early reports of alleged success in representing structure data, it has become clear that even deep learning methods do not provide a reliable representation of the data space [70]. This can also be seen in the “Hepta” example data set (Fig. 14C). The figure shows the original data set along with a similar sized data set generated using the ESOM-based model proposed here. The two data sets look alike (Fig. 14A and B). Autoencoders use supervised learning multilayer feedforward artificial neuronal networks (ANNs) to extract the essential features of the structure of a data set, which reduces its dimensions and can therefore be used for data projection. Autoencoders then learn to reconstruct the original data with the reduced representation. If the data set has a certain structure, this would be learned and emphasized in the reconstructed data. The training of an ANN was done with the goal of “identity,” i.e. all case vectors used as input to the autoencoder are reproduced identically as its output [71]. The neurons compute the logistic sigmoid function applied to the scalar product of the preceding neurons and the intermediate synoptic weights. The learning method used was backpropagation, a common implementation in autoencoders [72]. The network consisted of *n* = 3 input and *n* = 3 output neurons to represent the original “Hepta” data, which is 3D. Three hidden layers of five, three, and five neurons were used. After training the ANN, the central two neurons represent a 2D projection of the *n*-dimensional input data space, which is then used to reconstruct the data into the 3D output data space. Figure 14C shows the results, which are quite different from the original or the structure reconstructed using the ESOM generative algorithm.

On the other hand, a GMM gave satisfactory results for the “Hepta” data set (Fig. 14D). Specifically, GMMs are probabilistic models that assume that all data points are generated from a mixture of a finite number of Gaussian distributions with initially unknown parameters. The EM algorithm [73] can be used in GMMs to optimize the maximum likelihood; however, implementations of alternatives such as Markov chain Monte Carlo are available (summarized in Lötsch *et al*. [74]). Multivariate Gaussian uses a given multivariate normal distribution representing a *d*-dimensional mean vector. Fitting such a model to the “Hepta” data set provided the means and associated covariance matrix. This model was then successfully used to generate new data that fit the original data well (Fig. 14D). The results changed when the data set did not follow a Gaussian distribution, such as the three variables of the “Chainlink” dataset of the FCPS (Fig. 14F). Here, a GMM failed and its generative use produced data that bore little resemblance to the original data structure of the data set (Fig. 14I), while an autoencoder was quite successful in producing data that fit the original data well (Fig. 14H) and the new ESOM-based method provided again the expected satisfactory result (Fig. 14G).

The AI model generates data by exploiting multivariate structures identified by an ESOM of artificial neurons, inheriting limitations such as sensitivity to initialization parameters and constraints from the underlying generative model. The performance of the model depends on the ability of the ESOM to represent the data topology and generate realistic patterns. Data structures such as clusters can be defined by distance or density using methods such as *k*-means [75, 76] and density-based spatial clustering of applications with noise [77], respectively. In ESOM, the U-matrix captures distance-based structures, while the P-matrix addresses density-based structures. Although ESOM performed well on the mixed projection and clustering problems used in this report, it may struggle with datasets that combine distance- and density-based structures, which requires visual control of the projection results on the trained ESOM, as discussed in Lötsch and Ultsch [46]. In addition, the current AI paradigm is limited in generating data classes that are not present in the original sample. For example, if blue cells are very rare and yellow cells are absent in authentic data, AI cannot generate blue or yellow cells if they are not observed in a limited sample. This generative AI thus extrapolates from salient observations within a limited dataset while maintaining a low probability of hallucinating data, a phenomenon associated with large language models using other generative AI techniques than the present implementation [78]. Limitations on applicability are not the dimensionality of the data, which is handled by projection, nor the size of the data set, which results in fewer learning epochs. The ESOM requires rationally scaled data and is less suitable for nominal or ordinal scaled data, although, with the standard recording, such data are possible to process.

In this paper, we describe how generative learning can be achieved using ESOMs. Irrespective of the specific architecture, however, generative models share key features. That is, they all address the complex challenge of generating valid data from complex, often high-dimensional distributions that are either unknown or difficult to describe analytically. These models provide solutions to critical problems such as the paucity of rare cases, the reduction of extensive empirical testing (including animal testing), and the generation of data for meta-analysis. Several statistical approaches have been proposed for generative models, with rejection sampling, the Metropolis–Hastings algorithm, and the inverse transformation method being among the most prominent. When dealing with multivariate measures and their associated disease classifications (class labels), a fundamental question arises: does the class structure manifest itself in the numerical data in a way that allows meaningful disease-relevant analysis? In data science, this means determining whether the distance metric defined for the multivariate vectors is suitable for classification, clustering, and inference tasks. Moreover, the process of class labeling can be resource intensive [7], often resulting in only a small subset of data being labeled. To address these challenges, neural networks capable of data generation have gained popularity [21, 27]. These models differ from traditional classification learning approaches that aim to find the conditional distribution $p\left(c|x\right)$ and build classifiers with strong generalization performance, such as deep learning models [79]. Instead, generative learning focuses on finding the joint distribution $p\left(x,c\right)$. This joint distribution can then be used to generate previously unseen values of *x* and make class predictions *c* for these new data points [20, 27]. The power of generative models extends beyond the mere classification of existing data; they are distinguished by their ability to synthesize novel, authentic information that adheres to the patterns learned from the training data. This dual function - analysis and creation - distinguishes generative models as versatile tools in AI and machine learning. This capability is particularly valuable in scenarios where data are scarce, skewed, or difficult to obtain. In addition, generative models can provide insight into the data’s underlying structure, potentially revealing patterns and relationships that may not be apparent using other analytical methods. The present generative AI is embedded in the larger variety of generative AI models including recent advances in GANs, which have demonstrated their ability to generate data from minimal input [80–84]. While this report does not focus on GANs, their usefulness compared to the above methods (ESOM, GMM, autoencoder) was evaluated on an architecture called “generative adversarial training for synthesizing tabular data” (TGAN) [85]. The Python code was downloaded from https://github.com/sdv-dev/TGAN and run in a Python 3.7.16 environment on the two example datasets used above. The results support that GANs provide a suitable way to augment data sets from the current perspective of structure preservation. Specifically, both the FCPS “Hepta” and “Chainlink” datasets could be augmented with GAN-generated data in such a way that the augmented datasets preserved the original 3D structure well (Fig. 14E and J). The newer version of the tabular GAN-based data generator, called “CTGAN” [86] (https://github.com/sdv-dev/CTGAN), produced similar results in less time, especially when running on an NVIDIA GeForce RTX 4070. As the results did not differ from the TGAN version, they are not discussed further here. A comparison of all generated data shows that TGAN sticks (except for a few outliers) to the original structure of the “Chainlink” data set (see Fig. 14J). Structure detection and extrapolation based on ESOM allow for a sensible expansion of the variance for the data set, without intermixing of the group/cluster structures of the data. This can also be seen in the “PsA” data shown in Fig. 13, as discussed above.

All the method development efforts reported here reflect the need to augment small datasets when additional data are not available and highlight the importance of developing generative AI for numerical tabular research data as an active research area. Among the advantages of the present method is the accumulated evidence of its structure detection capabilities in small datasets, along with a publication record of applications to biomedical datasets [38, 40, 61, 64, 87] of the kind often encountered in laboratory research where data are limited and where these generative methods are therefore particularly needed.

## Conclusions

We propose a model-free generative algorithm based on unsupervised learning neural networks, specifically SOMs. It uses structure detection in multivariate data sets and identifies two distinct regions in (hyper)space: voids containing virtually no data points and dense data clouds. Within data clouds, the distances between neighboring data points (topological neighbors) are significantly smaller than in void regions. Through unsupervised learning, the neural network captures and represents the intrinsic structures of multivariate data spaces by detecting the cloud/void structures, without relying on a priori knowledge. We demonstrate the operation of this generative algorithm on several synthetic and biomedical datasets, including cases with small subgroups that exemplify rare disease settings or otherwise limited research data. After detecting the distance and density structures of the datasets using the U-matrix and P-matrix methods, respectively, a radius is obtained that defines the boundary of valid class structure and neighborhood-preserving generation of new data points. The generated data follow the distribution of the original data and cannot be discriminated from the original data by trained classification algorithms. The novel ESOM-based generative algorithm represents a promising solution for increasing sample sizes in small or rare case datasets, even when limited training data are available. This approach can address the challenges associated with small sample sizes in biomedical research, providing a tool to improve the reliability and robustness of scientific findings in this field.

**Key Points**

- Small sample sizes and rare cases hinder clinical translation and analysis in medical research.
- A novel unsupervised generative algorithm uses emergent self-organizing maps to detect structure in small multivariate datasets.
- The algorithm divides data into dense “cloud” and empty “void” regions and generates new points based on neighborhood relations.
- It successfully increases small group sizes while preserving original data structures without artifacts.
- It outperforms autoencoders and Gaussian mixture models on some datasets, providing an alternative to generative adversarial networks.

## Data Availability

All datasets were obtained from public sources that are accurately referenced in the report, with the exception of the psoriatic arthritis (“PsA”) dataset. This dataset is available from the appropriate authors upon reasonable request with a description of the purpose of use and pending approval by our local ethics and data safety review board.

## Code availability

The relevant script is part of this report and extends our R library “Umatrix” (https://cran.r-project.org/package=Umatrix).
